# Association Between Urinary Cotinine and Whole-Slide Digital Cytomorphometric Alterations in the Oral Mucosa of Tobacco Smoke-Exposed Cats

**DOI:** 10.3390/vetsci13040354

**Published:** 2026-04-04

**Authors:** Ilaria d’Aquino, Lorenzo Riccio, Giuseppe Piegari, Nicola Ambrosio, Consiglia Longobardi, Roberto Ciarcia, Laura Cortese, Evaristo Di Napoli, Orlando Paciello, Valeria Russo

**Affiliations:** 1 Unit of Pathology, Department of Veterinary Medicine and Animal Production, University of Naples “Federico II”, 80137 Naples, Italy; ilaria.daquino@unina.it (I.d.); evaristo.dinapoli@unina.it (E.D.N.); paciello@unina.it (O.P.); valeria.russo@unina.it (V.R.); 2 ASL Caivano, 80023 Naples, Italy; nicola.ambrosio@aslnapoli2nord.it; 3 Unit of Pharmacology, Department of Veterinary Medicine and Animal Production, University of Naples “Federico II”, 80137 Naples, Italy; consiglia.longobardi@unina.it (C.L.); rciarcia@unina.it (R.C.); 4 Unit of Internal Medicine, Department of Veterinary Medicine and Animal Production, University of Naples “Federico II”, 80137 Naples, Italy; lcortese@unina.it

**Keywords:** cotinine, precancerous stage, tobacco smoke exposure, oral malignant disorders, computational pathology, automated morphometric analysis

## Abstract

Cigarette smoke contains a high concentration of carcinogenic substances to which smokers are regularly exposed. Passive smoking is seriously harmful to the health of non-smoking humans and animals. Domestic cats are particularly vulnerable because of their constant grooming activity, which can promote oral ingestion of smoke-derived residues. Cotinine, a nicotine metabolite, is a reliable biomarker for tobacco exposure. Considering these observations, our study aimed to (1) characterize cytological alterations in oral mucosal epithelial cells by conventional morphology and automated digital cytomorphometry; (2) quantify urinary cotinine concentration and investigate its possible correlation with oral epithelial cytological alterations. To this aim, oral smears were collected from 30 cats divided into two groups (20 exposed; 10 non-exposed). Smears were stained with May–Grünwald–Giemsa and Papanicolaou to assess inflammation and dysplasia; digital cytomorphometric analysis was used to quantify the nucleus-to-cytoplasm (N/C) ratio. Urinary cotinine was measured by ELISA. Our results showed that exposed cats had significantly higher urinary cotinine levels and higher N/C ratios (*p* < 0.01) than non-exposed controls, along with mild-to-severe inflammation and dysplastic-like epithelial alterations. These findings support urinary cotinine as a valid biomarker of household tobacco smoke exposure in domestic cats and suggest that such exposure may be correlated with early cytological and cytomorphometric changes in the oral mucosa. Further studies are needed to better investigate the relationship between exposure duration and cytological, cytomorphometric, and molecular alterations.

## Simple Summary

Cigarette smoke comprises a variety of carcinogenic compounds that pose significant dangers to smokers as well as those subjected to passive tobacco smoke. Cats, which spend nearly half their day grooming, are especially vulnerable to ingesting smoke residues in indoor environments. Cotinine, a metabolite of nicotine, serves as a dependable biomarker for assessing tobacco exposure. This study aimed to characterize cytological and cytomorphometric alterations of the oral mucosal epithelium using morphological evaluation and automated digital image-assisted morphometry, and to quantify urinary cotinine concentration, investigating its possible correlation with oral epithelial cytological alterations. To this aim, oral smears were collected from 30 cats, stained with May–Grünwald–Giemsa and Papanicolaou dyes to assess inflammation and dysplasia; an automated cell analysis was performed to quantify the nucleus-to-cytoplasm (N/C) ratio. Cotinine concentrations in urine were determined using an ELISA-based immunoassay. Our results highlighted that exposed cats showed significantly higher urinary cotinine levels and increased N/C ratios, along with mild to moderate inflammation and epithelial dysplasia. These results indicate that cotinine in the urine is a valid biomarker of smoking exposure in domestic cats, and SHS can induce early morphological effects in oral mucosal epithelial cells.

## 1. Introduction

Cigarette smoke contains a high concentration of carcinogenic substances to which smokers are regularly exposed, such as nitrosamines, aromatic amines, phenols, and aldehydes [1]. Both smokers and non-smokers are exposed to these toxic irritants [2]. Active smokers inhale mainstream smoke (MSS), whereas passive smokers are exposed to secondhand smoke (SHS) [3]. Secondhand smoke is the involuntary inhalation of the sidestream smoke, produced by the combustion of a cigarette between puffs, and the exhaled mainstream smoke of the smoker [4]. SHS exposure reaches higher concentrations indoors, leaving toxic residues on surfaces of the place where it is smoked; these substances can persist long after the smoke has been removed from the environment. This phenomenon is known as thirdhand smoke (THS); THS exposure results from the involuntary inhalation, ingestion, or dermal uptake of THS pollutants in the air, in dust, and on surfaces, and it is a potentially dangerous condition, especially for children and domestic animals, who in addition to inhaling the smoke released by the cigarette, can also ingest the residues that end up on their hands or paws after touching the contaminated surfaces [5,6].

Regardless of whether the exposure is active or passive, in humans, nicotine is primarily metabolized in the liver, where it is converted approximately 70–80% to cotinine. Nicotine, cotinine, and other metabolites are excreted in urine [7,8]. Therefore, in humans, urine concentrations of these metabolites have been extensively used as biomarkers [9]. Several techniques have been validated to assess exposure to tobacco smoke; among them, cotinine level measurement is a reliable marker of tobacco smoke exposure due to its long half-life [8,10,11]. As in humans, similar metabolic pathways have been described in cats [6], where passive tobacco smoke exposure, such as tobacco smoke residues accumulated on the coat, may be ingested during grooming, resulting in repeated contact of toxicants with the oral mucosa. However, only a few studies have documented the toxicological and morphological impacts of passive tobacco smoke exposure and nicotine oral ingestion during grooming in cats [12,13,14]. Pathological effects of tobacco exposure are seriously harmful to the health of non-smoking humans and animals [15,16,17]. Indeed, in human medicine, even passive exposure is shown to induce precancerous and malignant oral cavity lesions [18,19,20], while in feline medicine, exposure to passive tobacco smoke has been associated with increased risk of malignant lymphoma [10,21,22] and oral squamous cell carcinoma [12,23]. Indeed, nicotine contributes to carcinogenesis by stimulating pro-proliferative and anti-apoptotic signaling pathways and inducing epigenetic changes, such as alterations in DNA methylation, histone modifications, and microRNAs, that modify gene expression and promote carcinogenesis [24].

Household pets are highly susceptible to passive tobacco smoke because they have close contact with human attendants and regular grooming activity that allows the body burdens of smoke residues stored in their hair coats to be ingested [25]. This double-exposure route, with both the lung [16] and oral routes potentially involved, is likely to increase local and systemic burdens of toxins. However, non-invasive tools to detect early smoke-associated epithelial changes in the feline oral cavity remain limited.

In this context, oral exfoliative cytology represents a minimally invasive diagnostic technique for detecting early epithelial alterations in the oral mucosa, eliminating the need for more invasive procedures, such as biopsy [26,27,28]. Moreover, in human medicine, the evaluation of cytomorphometric parameters is a valuable diagnostic tool [26,29,30,31].

Epithelial dysplasia is characterized by both architectural and cytologic alterations that reflect a progressive loss of normal cell proliferation and differentiation [32]. These cellular changes affect both the nucleus and the cytoplasm and include increased nucleus-to-cytoplasm (N/C) ratio, nuclear hyperchromasia, multinucleation, reduced cytoplasmic volume, and pleomorphism, which indicate a progressive loss of differentiation and regulatory proliferative control. Within this context, digital cytology and computer-assisted image analysis can support a more objective and reproducible quantification of cytomorphometric parameters [33,34], including the nuclear-to-cytoplasmic (N/C) ratio, a key indicator of early epithelial dysplasia.

Considering these observations, our study aimed to (1) characterize oral epithelial cytological alterations using morphological assessment and automated digital image-based morphometric analysis and (2) quantify urinary cotinine concentration and investigate its possible correlation with oral epithelial cytological alterations.

To date, no study has quantitatively integrated systemic exposure biomarkers with automated whole-slide digital cytomorphometry to assess early smoke-associated epithelial alterations in cats.

## 2. Materials and Methods

### 2.1. Ethical Statement

The study did not require consent or ethical approval under European Directive 2010/63/EU, as it falls under the exclusions set out in Article 2(2) of Italian Legislative Decree No. 26/2014. However, the study was evaluated by the University of Naples Federico II Animal Welfare Body/Ethical Animal Care and Use Committee on 28/06/2023 and received a favourable opinion (Protocol No. PG/2023/0114253, 25 September 2023). Written informed consent was obtained from all owners prior to enrollment, sample collection, and use of anonymized data for research and publication purposes.

### 2.2. Sample Collection and Inclusion Criteria

Cytologic smears from the oral cavity and urine samples were collected from 30 cats. Inclusion criteria were the absence of macroscopic oral lesions at the clinical examination, an age range between one and five years old, and an exclusively indoor lifestyle. Cat owners were interviewed using a written questionnaire to assess cats’ exposure to household tobacco smoke. Questions concerned the number of smokers in the household, the packs of cigarettes smoked per day at home, the number of years that each person smoked, and the proportion of time (per 24-h day) spent by the cat inside the home. Cases were divided into two groups based on the owners’ responses: Group A: cats with smoker owners (exposed); Group B: cats with non-smoker owners (control).

### 2.3. Morphological Analysis

The cytologic smears were collected from the oral mucosa of 30 cats using an oral brush; the brush was gently scraped across the oral mucosa to collect superficial epithelial cells. Each sample was smeared on three slides (n = 90) and fixed in 96% ethanol for 30 min. Two fixed smears were stained with MGG Quick stain (Bioptica, Milan, Italy); one smear was also stained with Papanicolaou stain (W01030708, Bioptica, Milan, Italy) for the evaluation of epithelial dysplasia, such as binucleated cells, cytoplasmatic chromatic alteration and abnormal keratinization. The Papanicolaou smear was immediately fixed by immersion in 95% ethanol. The samples were immersed in distilled water for about 2 min, followed by 1 min in Harris hematoxylin. This was followed by five minutes of prolonged washing in running water. The slides were then immersed in 95 per cent ethanol for 15 s to begin removing non-specific dyes, after which they were immersed in OG-6 solution for two minutes. A double step in 95% ethanol, lasting 15 s each, was then performed to fix the colour and prepare the slides for the second cytoplasmic dye. Smears were then immersed in EA-65 for 5 min. A series of ethanol rinses was performed: two changes in 95% ethanol with brief immersions of about 10 s each, followed by two changes in absolute ethanol (100%) lasting 1 min each, to complete dehydration. Finally, the slides were clarified in xylene (two changes of one to two minutes) and mounted. Oral inflammation was scored on a 0–3 scale (0 = no inflammation; 1 = mild; 2 = moderate; 3 = marked) as described by [35,36], based on inflammatory cells per high-power field (HPF) (<30, 30–100, and >100 inflammatory cells/HPF for mild, moderate, and marked inflammation, respectively); while Papanicolaou-stained smears were graded according to Kedra et al. [37].

### 2.4. Digital Cytomorphometric Analysis

All cytological slides were digitized using a 3DHISTECH whole-slide scanner at 40× magnification (0.14 µm/pixel), generating whole-slide images (WSI) in mrxs format [38]. This digitization enabled a standardized workflow for objective computation of the nucleus-to-cytoplasm (N/C) ratio. Digital cytomorphometric analysis was performed exclusively on May–Grünwald–Giemsa (MGG)-stained smears to ensure stain homogeneity for automated segmentation. The dataset comprised 60 MGG WSIs from 30 cats (two MGG slides per cat). Each WSI covered the entire cytological smear and was subdivided into 256 × 256 pixel tiles using a standardized computational tiling procedure consistent with established WSI analysis approaches [34]. A tissue mask was generated to exclude background regions, and candidate tiles were sampled from tissue-containing regions. To ensure morphometric measurements were obtained strictly from informative epithelial fields and to eliminate preservation artefacts, a rigorous supervised selection protocol was implemented. Candidate tiles were visually screened by an expert observer to retain only those containing at least one intact, clearly defined nucleated epithelial cell. Acellular fields, cellular overlaps, debris, inflammatory aggregates, and cells with incomplete cytoplasmic contours were strictly excluded. A minimum of 250 valid epithelial tiles per WSI were collected for the final dataset. A fixed number of 250 epithelial tiles per WSI was analysed, with one epithelial cell quantified per tile (250 cells/WSI). Selected tiles were processed using an automated image-analysis workflow to generate nuclear and cytoplasmic compartment masks. Briefly, tiles were transformed into CIELAB colour space and segmented using K-means clustering [39] to distinguish nuclear-enriched from cytoplasm-enriched regions. Binary masks were then refined using standard morphological operations and connected-component analysis to isolate individual epithelial cells and their corresponding nuclear and cytoplasmic compartments. Segmentation was performed using identical settings for all WSIs and visually checked on a representative subset of tiles to confirm accurate nuclear and cytoplasmic boundary delineation. Tile selection and quality-control followed predefined criteria applied uniformly across groups; while intra-observer reproducibility was assessed by having the same observer repeat tile selection on a random subset of WSIs using the same predefined criteria. For each segmented cell, the N/C ratio was calculated as:

N/C = A_nucleus/A_cytoplasm

For each cat, a case-level N/C ratio was computed as the median N/C ratio across all segmented epithelial cells from the two MGG WSIs.

### 2.5. Cotinine Assay

Urine samples from 21 cats (13 exposed; 8 non-exposed) were appropriately diluted and analysed using Cotinine ELISA (Enzyme-Linked Immunosorbent Assay) kit (Abnova, Taoyuan City, Taiwan). Briefly, 100 uL of the HRP (Horseradish peroxidase) enzyme conjugate was added to each well and incubated for 60 min at r.t. in the dark. After incubation, wells were washed three times, inverted, and tapped vigorously on adsorbent paper to remove excess liquid. Then, 100 µL of substrate reagent was added to each well and incubated at room temperature in the dark. After a few minutes, 100 uL of stop solution was added simultaneously to all wells by multichannel pipette, and absorbance was immediately measured at 450 nm using a spectrophotometer (ThermoFisher, Carlsbad, CA, USA). A positive and negative control were included in each assay. Cotinine concentrations were expressed as ng/mL of cotinine based on a standard curve. According to the manufacturer, the assay detection range was 0–100 ng/mL, with a limit of detection (LOD) of 1 ng/mL. The intra- and inter-assay coefficients of variation were within the ranges typically reported for ELISA assays.

### 2.6. Statistical Analysis

Group comparisons (exposed vs. non-exposed) were performed using two-sided Mann–Whitney U tests, with effect size quantified by Cliff’s delta. Categorical variables (sex) were compared using Fisher’s exact test, with effect size reported as odds ratio (OR) with 95% confidence intervals. Associations among urinary cotinine, N/C ratio, inflammation score, dysplasia grade, and age were assessed within the exposed cohort using Spearman’s rank correlation. For effect sizes and coefficients, 95% confidence intervals were computed using standard methods (Cliff’s delta: nonparametric bootstrap; OR: exact 95% CI; Spearman’s ρ: approximate 95% CI based on Fisher’s z-transformation). All tests were two-sided (α = 0.05). Analyses were performed in Python 3.11 using the following libraries: NumPy 2.4.2, pandas 3.0.1, and SciPy 1.17.1.

## 3. Results

### 3.1. Study Population and Baseline Characteristics

A total of 30 indoor cats were enrolled (n = 20 exposed; n = 10 non-exposed). Individual demographic and exposure data are reported in Table 1, while baseline group comparisons are summarized in Table 2. Age and sex distribution did not differ between groups (both *p* ≥ 0.05), although non-exposed cats tended to be older (median 5.0 vs. 1.5 years). Exposure stratification was confirmed by significant differences in both daily cigarette consumption and number of smoker owners (both *p* < 0.001). In exposed households, the median cigarette consumption was 8.5 cigarettes/day (Table 2). Regarding the number of active smokers per household, 80% of exposed cats (16/20) lived with one smoker and 20% (4/20) lived with two smokers (Table 1).

### 3.2. Morphological Evaluation

**Figure 1:**
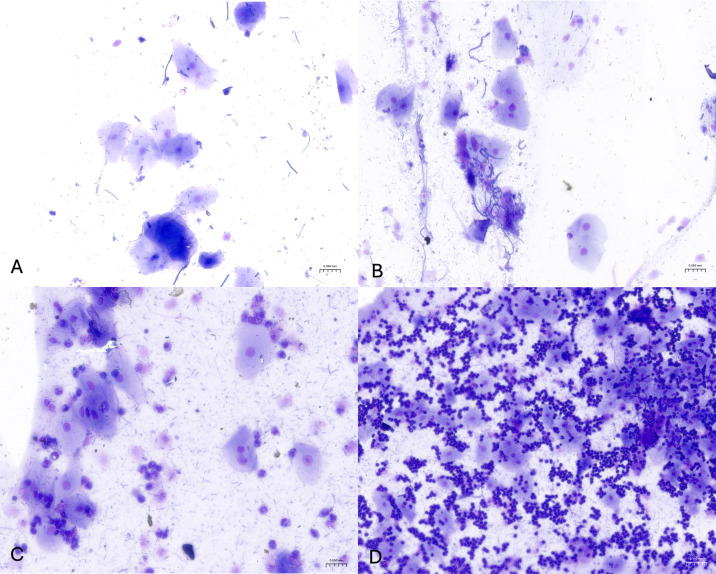
Representative May–Grünwald–Giemsa-stained oral cytological smears showing different grades of inflammation: (**A**) absence of inflammation (score 0); (**B**) mild inflammation (score 1); (**C**) moderate inflammation (score 2); (**D**) marked inflammation (score 3). (**A**,**C**) Original magnification: 20×. (**D**) Original magnification: 10×.

**Figure 2:**
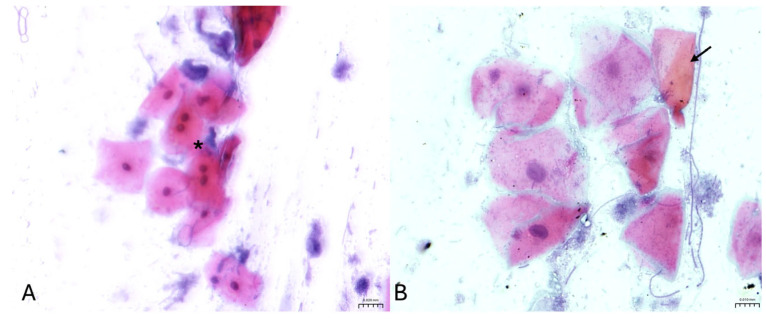
Representative Papanicolaou-stained oral cytological smears showing dysplastic-like features, including cytoplasmic tinctorial shifts (green to pink), orangeophilic keratinized cytoplasm (arrow), and binucleation (asterisk). (**A**) Original magnification: 40×. (**B**) Original magnification: 60×.

**Figure 3:**
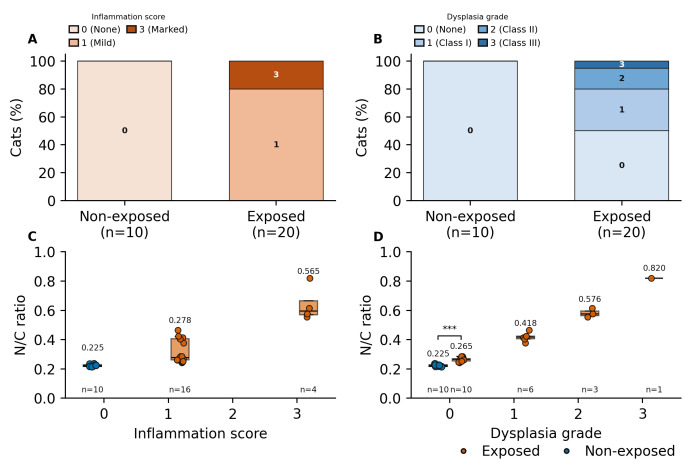
(**A**) Distribution of inflammation scores. (**B**) Distribution of dysplasia grades. (**C**) N/C ratio by inflammation score. (**D**) N/C ratio by dysplasia grade. Points indicate individual cats (exposed, orange circles; non-exposed, blue circles); boxes show median and IQR, whiskers indicate 10th–90th percentiles; *** *p* < 0.001.

Oral brush smears stained with May–Grünwald–Giemsa (Figure 1) showed variable degrees of inflammation in exposed cats. Specifically, 4/20 exposed cats (20%) showed marked inflammation (score 3), characterized by numerous viable and degenerate neutrophils, with occasional macrophages, lymphocytes, and plasma cells. The cytological background was moderately detrital, with proteinaceous material and bacteria. The remaining 16/20 exposed cats (80%) showed mild inflammation (score 1), characterized by a low number of neutrophils with rare lymphocytes and plasma cells; the background similarly appeared moderately detrital, with proteinaceous material and bacteria. No cases with moderate inflammation (score 2) were observed. In contrast, 10/10 non-exposed cats (100%) showed no inflammation (score 0), with an almost complete absence of inflammatory cells and a clean background consisting predominantly of epithelial cells. On Papanicolaou-stained smears (Figure 2), 10 of 20 exposed cats (50%) showed dysplastic-like changes. Dysplasia grades were distributed as follows: class I in 6/20 cats (30%), class II in 3/20 cats (15%), and class III in 1/20 cats (5%). Dysplastic changes included cytoplasmic staining shifts (green-to-pink), orange keratinized cytoplasm, and binucleation. The remaining 10/20 exposed cats (50%) showed no cytological signs of dysplasia. All non-exposed cats (10/10; 100%) also showed no cytological signs of dysplasia. Automated morphometric analysis revealed a clear separation in nucleus-to-cytoplasm (N/C) ratio between groups and across lesion severity (Figure 3). The distributions of inflammation scores and dysplasia grades in non-exposed and exposed cats are summarized in Figure 3A,B, respectively. Non-exposed cats clustered at the lower end of the N/C ratio distribution (median 0.225 (Q1–Q3: 0.218–0.227); range 0.215–0.236), whereas exposed cats showed a broader range extending to higher values (range 0.242–0.615) (Figure 3C,D). Among cats without dysplasia (grade 0), exposed cats showed higher N/C ratios than non-exposed cats (median 0.265 (Q1–Q3: 0.254–0.271) vs. 0.225 (Q1–Q3: 0.218–0.227); Mann–Whitney U test, *p* < 0.001) (Figure 3D). In exposed cats with dysplasia, N/C ratios were higher in grade 1 and grade 2 compared with grade 0 (grade 1: median 0.418 (Q1–Q3: 0.406–0.423), n = 6; grade 2: median 0.576 (Q1–Q3: 0.565–0.596), n = 3). The single grade 3 case showed an N/C ratio of 0.820 (n = 1) (Figure 3D). A similar pattern was observed for inflammation severity, with exposed cats showing higher N/C ratios in mild inflammation (score 1: median 0.278 (Q1–Q3: 0.261–0.406), n = 16) and marked inflammation (score 3: median 0.565 (Q1–Q3: 0.545–0.586), n = 4) compared with non-exposed cats (score 0: median 0.225 (Q1–Q3: 0.218–0.227), n = 10) (Figure 3C).

### 3.3. Urinary Cotinine Concentrations

**Figure 4:**
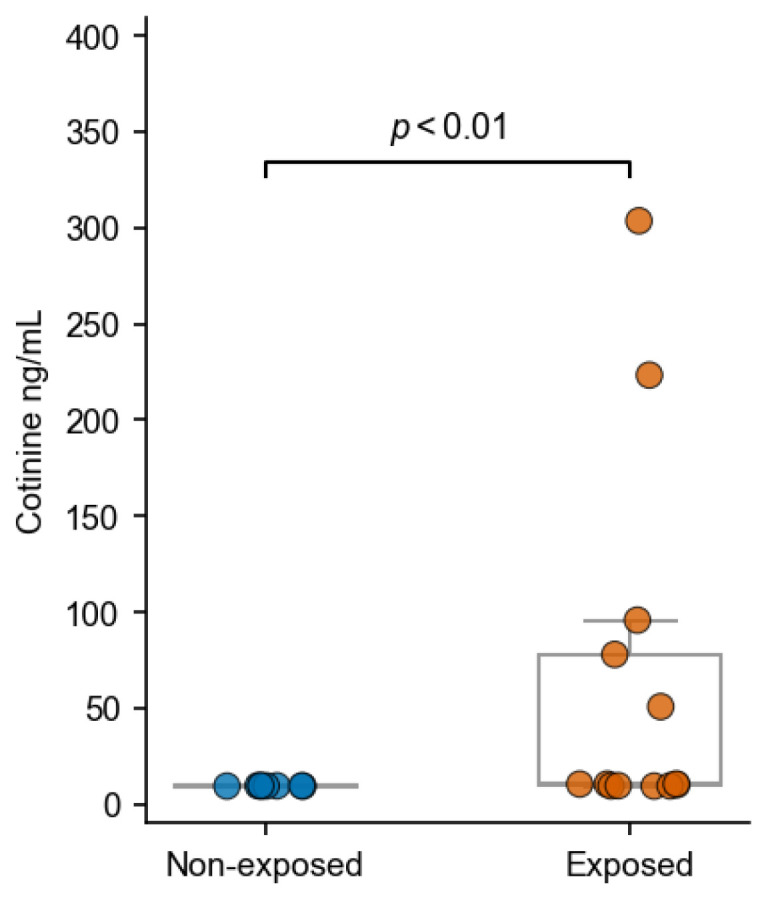
Urinary cotinine concentrations in exposed and non-exposed cats. Scatter plot of urinary cotinine (ng/mL; ELISA) in smoke-exposed (n = 13) and non-exposed controls (n = 8). Each point represents one animal. Cotinine concentrations were significantly higher in exposed cats than in controls (*p* < 0.01, Mann–Whitney U).

Urinary cotinine concentrations were significantly higher in smoke-exposed cats compared to non-exposed cats (*p* < 0.01; Figure 4), confirming a separation between the two groups. In detail, within the exposed cohort (n = 13), cotinine values showed marked inter-individual variability, consistent with heterogeneous household exposure conditions. Most exposed cats showed low to moderate concentrations, whereas a distinct subset (2/13) exceeded 200 ng/mL, indicating substantially higher nicotine uptake in these individuals. In contrast, cotinine levels in non-exposed cats (n = 8) remained consistently low and clustered near baseline.

### 3.4. Association Between Urinary Cotinine and N/C Ratio

**Figure 5:**
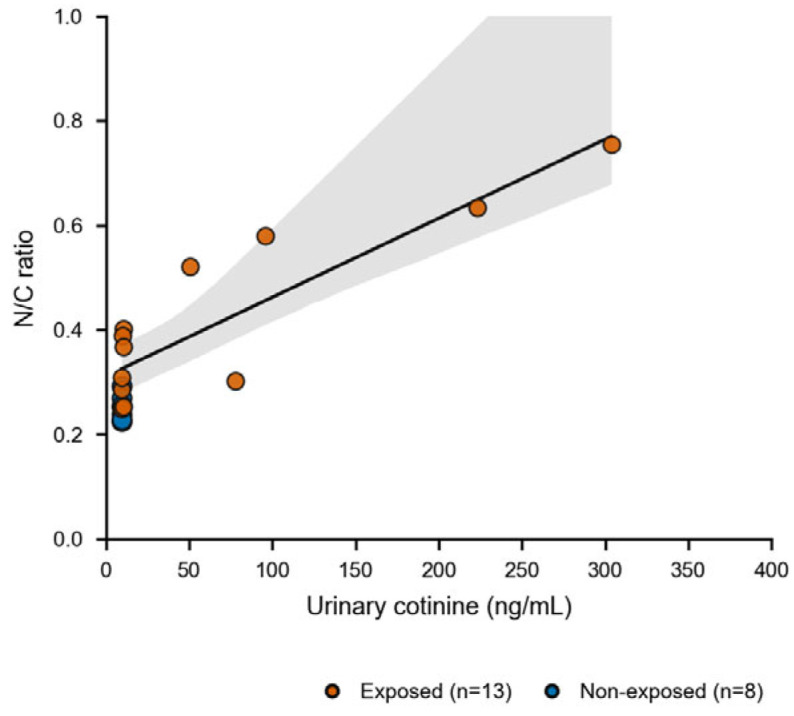
Urinary cotinine levels vs. N/C ratio. Scatter plot of animal-level N/C ratio versus urinary cotinine concentration; symbols indicate exposure group. The fitted line and shaded band are shown for visualization only.

Automated morphometric analysis showed lower N/C ratios in non-exposed controls, which clustered tightly at the lower end of the distribution, whereas exposed cats spanned a wider range. As shown in Figure 5, higher urinary cotinine levels were associated with higher N/C ratios, consistent with a dose-related increase in cytomorphometric damage. The cotinine–N/C association was tested within exposed cats with available urinary cotinine measurements using Spearman’s rank correlation (exposed only, n = 13; two-sided *p* < 0.001; ρ = 0.85; 95% CI [0.56–0.95]).

### 3.5. Correlation Analysis

**Figure 6:**
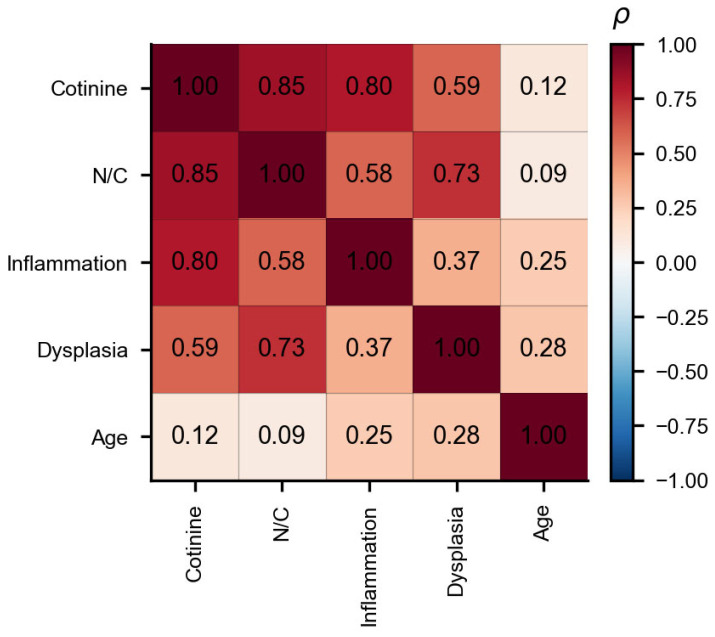
Spearman correlation matrix of study variables within the exposed cats with complete data. The heatmap displays Spearman’s rank correlation coefficients (ρ) among urinary cotinine, N/C ratio, inflammation score, dysplasia grade, and age.

Among the exposed cats with complete data on urinary cotinine, N/C ratio, inflammation score, dysplasia grade, and age (n = 13), Spearman’s rank correlation was used to examine the associations among the study variables (Figure 6). Urinary cotinine showed a strong positive association with the N/C ratio (ρ = 0.85, *p* < 0.001). Cotinine was also positively associated with inflammation score (ρ = 0.80, *p* < 0.001) and showed a moderate association with dysplasia grade (ρ = 0.59, *p* < 0.05). The N/C ratio was positively associated with dysplasia grade (ρ = 0.73, *p* < 0.01) and inflammation score (ρ = 0.58, *p* < 0.05), whereas the inflammation–dysplasia association was weaker and not statistically significant (ρ = 0.37, *p* ≥ 0.05). Age showed only weak, non-significant associations with the other variables (|ρ| ≤ 0.28, *p* ≥ 0.05).

## 4. Discussion

Domestic cats serve as unique sentinel species for indoor air quality assessment due to their intensive grooming behaviour and close cohabitation with humans, which exposes them to shared household pollutants [10]. The specific cytomorphometric effects of chronic household tobacco smoke on the feline oral mucosa remain incompletely quantified. Although associations between smoke exposure and oral neoplasia are well-documented in humans [29,30], objective evidence linking systemic exposure biomarkers to early cellular alterations in cats is limited. To address this gap, our study integrates urinary cotinine quantification with a standardized digital cytology workflow to objectively quantify cytomorphometric alterations in the oral mucosa of cats exposed to household tobacco smoke. Overall, our findings provide quantitative evidence for the hypothesis that chronic exposure to household tobacco smoke is associated with biological and cytomorphometric changes in the oral mucosa of domestic cats. Cats living in smoking households had significantly elevated urinary cotinine concentrations and elevated N/C ratios compared to non-exposed cats, supporting an association between passive tobacco exposure and early epithelial atypia and dysplastic-like cytological changes detected by oral exfoliative cytology [10,12]. The findings agree with previous studies that characterize the adverse effects of passive smoke for animals and humans [6]. In indoor environments, SHS and residual THS contamination may co-occur and jointly contribute to overall household tobacco smoke exposure [40,41]. Specifically, the right shift in the N/C-ratio distribution in exposed cats provides early cytologic evidence of dysplasia-like epithelial alteration, including nuclear enlargement and cytoplasmic reduction. Comparable morphometric patterns have been documented in human oral cytology related to tobacco exposure [29,30] and in feline oral squamous cell carcinoma [12]. The elevated N/C ratio identified by automated analysis may thus represent a sensitive early indicator of smoke-induced mucosal injury [33]. Whole-slide digital cytology combined with automated morphometry enabled a standardized, quantitative assessment of the N/C ratio, reducing subjectivity compared with conventional microscopic estimation and supporting cross-case comparability [33,34,42]. K-means color segmentation in CIELAB space provided a transparent, training-free approach for nuclear–cytoplasmic compartment separation, yielding consistent N/C estimation across WSIs [34,39]. Methodological robustness was further supported by fixed segmentation settings and predefined quality-control criteria applied uniformly across groups, and by case-level aggregation of measurements (median N/C ratio across two WSIs), which mitigates local smear heterogeneity and reduces the influence of extreme values [33,34]. In addition to morphometric findings, Papanicolaou-stained smears provided qualitative cytological evidence of exposure-associated epithelial alteration [20,43,44]. Dysplastic-like features were observed in 10/20 exposed cats but not in controls, including cytoplasmic tinctorial shifts (green-to-pink), increased orangeophilia suggestive of abnormal keratinization, and binucleation. Papanicolaou cytology was used here to describe epithelial atypia; however, confirmation and grading of dysplasia remain histology-based [20,43,44]. Inflammation was also observed in exposed cats, predominantly characterized by neutrophilic infiltration with occasional macrophages, lymphocytes, and plasma cells. This finding represents a biologically relevant cofactor, as chronic inflammatory microenvironments may promote epithelial instability through oxidative stress and cytokine-mediated pathways [45,46,47]. The correlation between cotinine levels and morphometric parameters suggests urinary cotinine acts as both an exposure biomarker and a biological response indicator [11,25]. Within exposed cats, urinary cotinine was correlated with the N/C ratio (ρ = 0.85, *p* < 0.001) and showed positive associations with inflammation score (ρ = 0.80, *p* ≤ 0.001) and dysplasia grade (ρ = 0.59, *p* < 0.05), suggesting that higher nicotine uptake is associated with more pronounced cytomorphometric alteration in this cohort. However, these correlations should be interpreted as exploratory, given the limited sample size and cross-sectional design. Our findings are consistent with human clinical research, where chronic exposure to cigarette smoke is associated with morphologic and morphometric changes such as nuclear hypertrophy, cytoplasmic shrinkage, and elevated N/C ratio. These features are typically used as indicators of early epithelial dysplasia and premalignant progression in oral cytology [29,30,48,49]. Evaluation of precancerous stages, including dysplasia-like changes, enables prevention and risk stratification before malignant transformation [20,43,44]. In this framework, N/C ratio shifts, evaluated together with inflammation and dysplasia grading and supported by urinary cotinine as a systemic exposure biomarker, may support follow-up prioritization and longitudinal monitoring in exposed animals [11,25]. Cats share the same indoor exposure sources as their owners, supporting their use as sentinels for household tobacco-smoke exposure. Within a One Health perspective, cross-species consistency with human tobacco-related oral cytology findings supports the translational relevance of these early morphometric changes [10,13,25,29,30]. This non-invasive framework combines systemic exposure biomarkers with WSI-based computational morphometry to support early risk stratification and longitudinal monitoring [10,13,25,29,30].

## 5. Limitations

A few limitations of the study should be noted. First, the sample size was limited and unbalanced between groups. Second, urine samples were not available for all enrolled cats and the exposure assessment relied on owner-reported data. Third, the difference in age distribution between exposed and non-exposed cats may have acted as a potential confounding factor. Finally, although morphometric analysis was performed using an automated digital workflow, manual tile selection was required before analysis and may therefore have introduced a degree of observer dependency. Despite these limitations, the integration of a systemic biomarker, qualitative cytological evaluation, and quantitative morphometry defines a scalable, non-invasive assessment workflow, offering a robust model for environmental toxicology and comparative veterinary pathology.

## 6. Conclusions

In our study, cats from households exposed to tobacco smoke showed higher urinary cotinine concentrations and increased case-level N/C ratios derived from automated whole-slide digital cytology, supporting an association between household tobacco smoke exposure and quantifiable oral epithelial alterations consistent with early atypia. In indoor environments, household tobacco smoke exposure may reflect combined contributions of secondhand smoke and residual thirdhand smoke contamination. These quantitative findings were consistent with exposure-associated qualitative cytological changes observed in Papanicolaou-stained smears, while recognizing that confirmation and grading of epithelial dysplasia remain histology-based. The automated WSI-based workflow enabled standardized region selection and objective morphometric quantification, reducing observer dependence relative to conventional microscopy and providing a scalable framework for non-invasive screening and monitoring in veterinary environmental health. This approach may support prevention and risk stratification through follow-up prioritization in chronically exposed cats. Because cats share the indoor microenvironment with their owners, these findings also support a One Health perspective and are consistent with smoke-associated morphometric patterns reported in human oral cytology. Further studies with larger cohorts, longitudinal follow-up, and complementary biomarkers of genomic damage are needed to better clarify temporal relationships and strengthen the link between tobacco smoke exposure and early cytomorphometric alterations.

## Figures and Tables

**Table 1:** Summary of the study population and household tobacco-smoke exposure status (Exposed vs. Non-exposed).

Cat ID	Exposure	Age	Sex	Smoker Owners (n)	Cigarettes/Day
1	Exposed	1	M	1	6
2	Exposed	2	M	2	10
3	Exposed	1	F	1	10
4	Exposed	5	M	1	10
5	Exposed	1	M	1	7
6	Exposed	1	F	1	10
7	Exposed	2	M	1	10
8	Exposed	5	M	1	5
9	Exposed	1	M	1	10
10	Exposed	5	M	1	10
11	Exposed	1	M	1	5
12	Exposed	5	M	1	7
13	Exposed	5	F	2	9
14	Exposed	5	M	2	11
15	Exposed	5	F	1	5
16	Exposed	1	M	1	7
17	Exposed	5	M	1	6
18	Exposed	1	F	2	9
19	Exposed	1	F	1	4
20	Exposed	1	F	1	8
21	Non-exposed	5	F	0	0
22	Non-exposed	5	M	0	0
23	Non-exposed	4	F	0	0
24	Non-exposed	1	F	0	0
25	Non-exposed	5	F	0	0
26	Non-exposed	5	M	0	0
27	Non-exposed	5	F	0	0
28	Non-exposed	1	M	0	0
29	Non-exposed	4	M	0	0
30	Non-exposed	2	F	0	0

**Table 2:** Baseline characteristics of enrolled cats (Exposed, n = 20; Non-exposed, n = 10). Continuous variables are reported as median [IQR] and compared using two-sided Mann–Whitney U tests, with effect size estimated by Cliff’s delta. Sex was analyzed as a binary variable and compared using Fisher’s exact test; effect size is reported as odds ratio (OR).

Variable	Exposed	Non-Exposed	Test	*p*-Value	Effect Size
Age (years)	1.50 [4.00]	4.50 [2.50]	Mann–Whitney U	*p* ≥ 0.05	Cliff’s d = −0.25 (95% CI [−0.61–0.14])
Smoker owners (n)	1.00 [0.00]	0.00 [0.00]	Mann–Whitney U	*p* < 0.001	Cliff’s d = 1.00 (95% CI [1.00–1.00])
Cigarettes/day	8.50 [4.00]	0.00 [0.00]	Mann–Whitney U	*p* < 0.001	Cliff’s d = 1.00 (95% CI [1.00–1.00])
Female	7 (35.0%)	6 (60.0%)	Fisher exact	*p* ≥ 0.05	OR = 0.359 (95% CI [0.08–1.71])
Male	13 (65.0%)	4 (40.0%)	Fisher exact	*p* ≥ 0.05	OR = 2.79 (95% CI [0.58–13.31])

## Data Availability

The original contributions presented in this study are included in the article. Further inquiries can be directed to the corresponding author(s).
